# Multiple Regulatory Levels Shape Autophagy Activity in Plants

**DOI:** 10.3389/fpls.2019.00532

**Published:** 2019-04-24

**Authors:** Mingkang Yang, Fan Bu, Wei Huang, Liang Chen

**Affiliations:** State Key Laboratory for Conservation and Utilization of Subtropical Agro-bioresources, College of Life Sciences, South China Agricultural University, Guangzhou, China

**Keywords:** autophagy, transcriptional regulation, RNA decay, protein modification, plant

## Abstract

Autophagy is a strictly regulated pathway involving the degradation of cytoplasmic organelles and proteins. Most autophagy-related genes have been identified in plants based on sequence similarity to homologues in yeast and mammals. In addition, the molecular mechanisms underlying plant autophagy have been extensively studied in the last decade. Plant autophagy plays an important role in various stress responses, pathogen defense, and developmental processes such as seed germination, pollen maturation, and leaf senescence. However, the regulatory mechanisms of autophagy in plants remain poorly understood. Recent studies have identified several plant autophagy regulators, which modify autophagy activity at transcriptional, post-transcriptional, and post-translational levels. In this review, we summarize recent advances in understanding regarding regulatory network of plant autophagy and future directions in autophagy research.

## Introduction

Autophagy is a highly conserved cellular process for the degradation of cytoplasmic organelles and long-lived proteins in eukaryotes. Two types of autophagy, microautophagy, and macroautophagy, have been identified in plants (Bassham et al., 2006). During microautophagy, cytoplasmic components are engulfed directly by the invagination of the tonoplast and then degraded by vacuolar hydrolases. In contrast, during macroautophagy, a double-membrane vesicle, called an autophagosome, delivers cytoplasmic materials to the vacuole for degradation. Here, we mainly focus on the macroautophagy (hereafter termed autophagy) in plants.

The autophagy process is mediated by a set of evolutionally conserved Autophagy-related (ATG) proteins, which was originally identified in yeast (Matsuura et al., 1997). Most ATG proteins function in autophagosome formation have also been identified in plants (Marshall and Vierstra, 2018). These proteins could be divided into four core functional groups: the ATG1 kinase complex involved in the initiation of autophagosome formation; the ATG9 complex for membrane recruitment; the phosphatidylinositol 3-kinase (PI3K) complex for vesicle nucleation; and ATG8 and ATG12 ubiquitin-like conjugation systems for vesicle expansion and closure (Liu and Bassham, 2012).

Plants have evolved intricate mechanisms to cope with various environmental stresses because of their immobility. Emerging evidence has indicated that autophagy is involved in their responses to biotic and abiotic stresses, such as nutrient deficiency (Doelling et al., 2002), oxidative stress (Xiong et al., 2007), salt stress (Luo et al., 2017), drought (Liu et al., 2009), hypoxia (Chen et al., 2015), and pathogen infection (Lai et al., 2011). However, the molecular mechanism of autophagy activated by environmental stresses remains largely unknown. Herein, we review recent advances in the understanding of the regulatory pathway underlying plant autophagy at the transcriptional, post-transcriptional, and post-translational levels.

### Transcriptional Regulation

At present, around 40 *ATG* genes have been isolated and identified by genetic screening of yeast, and homologs of many *ATG* genes have been characterized in plants (Marshall and Vierstra, 2018). The transcriptional levels of *ATG* genes are increased under various stress conditions in plants, including *Arabidopsis*, wheat (*Triticum aestivum*), rice, and tomato (Xia et al., 2011; Pei et al., 2014; Chen et al., 2015; Zhou et al., 2015). Transcriptional regulation is essential for the expression of *ATG* gene in response to environment stresses. Recently, some studies have identified a few transcriptional regulators that directly regulate *ATG* genes (Table 1).

**Table 1:** Transcriptional regulators of autophagy in plants.

Transcriptional regulators	Target genes	Effects	Species	References
HsfA1a	*ATG10, ATG18f*	Enhanced autophagy	*Arabidopsis thaliana*	Wang et al., 2015
WRKY33	Unidentified	Enhanced autophagy	*Arabidopsis thaliana*	Lai et al., 2011
	Unidentified	Enhanced autophagy	*Solanum lycopersicum*	Zhou et al., 2014
WRKY20	*ATG8a*	Enhanced autophagy	*Manihot esculenta*	Yan et al., 2017
BZR1	*ATG2, ATG6*	Enhanced autophagy	*Arabidopsis thaliana*	Wang et al., 2019
ERF5	*ATG8d, ATG18h*	Enhanced autophagy	*Solanum lycopersicum*	Zhu et al., 2018
HDA9	*ATG9*	Suppressed autophagy	*Arabidopsis thaliana*	Chen et al., 2016


#### Heat Shock Protein: HsfA1a

Heat shock proteins (Hsps) are produced in response to stresses and function by stabilizing or refolding proteins. Heat shock transcription factors (Hsfs), are the transcription factors that regulate the expression of stress-responsive genes, including genes encoding Hsps. Plant Hsfs are classified into three conserved evolutionary categories (HsfA, B, and C) according to the protein structure (Guo et al., 2016). HsfA1a is found to regulate autophagy in plants (Wang et al., 2015). Upon drought stress, tomato HsfA1a is induced and activated by trimerization. The activated form of HsfA1a directly binds to the heat-shock elements in the promoter of *ATG10* and *ATG18f*. In addition, the number of autophagosomes and transcript levels of *ATG10* and *ATG18f* are improved by *HsfA1a* overexpression but reduced by *HsfA1a* silencing under drought stress (Wang et al., 2015). Therefore, HsfA1a positively regulates autophagy and confers drought tolerance in tomato.

#### Transcription Factors: WRKY Family

WRKYs are a large family of transcription factors that modulate many plant physiological processes, such as growth, development, and response to abiotic and biotic stresses (Rushton et al., 2010). Some WRKY transcription factors induce expression of ATG genes under biotic and abiotic stresses (Lai et al., 2011; Zhou et al., 2014; Yan et al., 2017). WRKY33 regulates pathogen-induced and heat-induced autophagy in plants (Lai et al., 2011; Zhou et al., 2014). In *Arabidopsis*, a WRKY33 mutation results in the downregulation of the expression of *ATG18a*, an essential factor for autophagosome formation, and decreasing autophagic activity upon Botrytis infection (Lai et al., 2011). Moreover, WRKY33 interacts with ATG18a in the nucleus which indicates that ATG18a may self-regulate its own expression by acting as a co-factor with WRKY33 (Lai et al., 2011). These results suggest that WRKY33 plays a critical role in the positive regulation of pathogen-induced autophagy (Lai et al., 2011). WRKY33 also involves in regulation of heat-induced autophagy (Zhou et al., 2014). *ATG* gene expression and autophagosome accumulation are induced by heat stress in both tomato and *Arabidopsis* plants (Zhou et al., 2013, 2014). Suppression of autophagy leads to a decrease in the heat tolerance of tomato and *Arabidopsis* plants (Zhou et al., 2013, 2014). Silencing of tomato *WRKY33a* or *WRKY33b* decreases the expression of *ATG5* and *ATG7* and autophagosome formation, and compromises tomato heat tolerance (Zhou et al., 2014). Although WRKY33 has been indicated to be involved in autophagy regulation, the exact molecular mechanisms that underlie this regulation ramian unknown. WRKY20 is a transcriptional activator of *ATG8a* and is essential for disease resistance against bacterial blight in cassava (Manihot esculenta) (Yan et al., 2017). WRKY20 is upregulated upon infection of cassava bacterial blight, which is caused by *Xanthomonas axonopodis* pv. *manihotis* (Xam). Then, WRKY20 directly binds to the W-box in the promoter of *ATG8a* and activates its expression (Yan et al., 2017). On the other hand, WRKY20 directly interacts with ATG8 proteins indicating that WRKY20 is degraded by autophagy to form a feedback loop (Yan et al., 2017).

#### Transcriptional Factor in Brassinosteroid Pathway: BZR1

Brassinosteroids (BRs) play crucial roles in stress responses, growth, and development of plants (Krishna, 2003; Zhu et al., 2013). A recent paper reported that BRASSINAZOLE RESISTANT 1 (BZR1), a vital transcriptional factor in BR signal transduction, plays a positive role in the autophagy pathway (Wang et al., 2019). Brassinolide (BL) treatment promotes *ATG* gene expression and autophagosome formation. In addition, the BL-induced *ATG* gene expression and autophagosome formation are enhanced in *BZR1*-overexpressing plants and compromised in *BZR1*-silenced plants. Results of ChIP and yeast one-hybrid assays show that BZR1 directly binds to the promoters of *ATG2* and *ATG6* (Wang et al., 2019). These findings suggest that BZR1-mediated BR signaling positively regulates autophagy. On the other hand, BZR1is selectively degraded by autophagy (Zhang Z. et al., 2016). These results suggest that there is feedback regulation between BZR1-dependent BR signaling and the autophagy pathway.

#### Transcriptional Factor in Ethylene Pathway: ERF5

The ethylene pathway is involved in the regulation of autophagy (Okuda et al., 2011; Shibuya et al., 2013). In one of these studies, *ATGs* and ethylene-related genes were induced in soybean (*Glycine max*) by sugar and nitrogen starvation, and 1-aminocyclopropane-1-carboxylic acid (ACC, the precursor of ethylene) enhanced the expression of *ATG8i* (Okuda et al., 2011). Ethylene rapidly induced *ATG8s* expression, while ethylene inhibitor delayed the induction of *ATG8s* in petunia petals (Shibuya et al., 2013). ERF5 (ethylene response factor 5) is significantly induced by ACC and drought treatment while ERF5 overexpression confers high tolerance to drought in the tomato plant (Pan et al., 2012). Under drought stress, ERF5 directly binds to the promoters of *ATG8d* and *ATG18h* and activates gene expression to promote autophagy, which is essential for ethylene-mediated drought resistance (Zhu et al., 2018).

#### Histone Deacetylase: HDA9

Histone acetylation participates in transcriptional regulation of gene expression in eukaryotic cells (Struhl, 1998). Histone acetylation is usually associated with transcriptional activation. Conversely, deacetylation represses gene transcription. Histone acetylation levels are reversibly regulated by histone acetyltransferases and histone deacetylases (HDAs). HDA9 is shown to play an important role in autophagy-dependent leaf senescence (Chen et al., 2016). In *Arabidopsis*, HDA9 is transported from the cytoplasm into the nucleus by interacting with POWERDRESS (PWR). Together with WRKY53, HDA9 and PWR bind to W-box of the *ATG9* promoter. Furthermore, HDA9 and PWR mutations lead to the upregulation of the *ATG9* transcript by H3K27 hyperacetylation at *ATG9* genomic regions (Chen et al., 2016). These results indicate that PWR recruits HDA9 and WRKY53 at the W-box motif of the *ATG9* promoter to remove H3 acetylation marks, and then suppresses *ATG9* gene expression to promote leaf senescence.

### Post-transcriptional Regulation

Autophagy can be regulated at the post-transcriptional level by microRNAs (miRNAs) in animals (Feng et al., 2015). However, miRNA-mediated autophagy regulation has not been found in plants. Instead, autophagy is regulated at the post-transcriptional level through inositol-requiring enzyme-1 (IRE1)-dependent decay of mRNAs (RIDD) in *Arabidopsis* (Bao et al., 2018). IRE1 functions as both a kinase and a ribonuclease and was first identified as an ER stress sensor in yeast (Cox and Walter, 1996; Mori et al., 1996). IRE1 regulates ER stress-induced autophagy by ribonuclease splicing activity through the IRE-HAC1 (homologous to ATF/CREB 1) signaling pathway in yeast (Yorimitsu et al., 2006), but by kinase activity through the IRE1-JNK (c-Jun N-terminal kinase 1) pathway in animals (Ogata et al., 2006). There are two IRE1 homologs (IRE1a and IRE1b) responsible for the splicing of *bZIP60* mRNA in *Arabidopsis* (Koizumi et al., 2001; Nagashima et al., 2011). IRE1b was identified as a regulator of autophagy during ER stress in plants (Liu et al., 2012). However, the molecular mechanism underlying this process was unclear until recently when it was reported that IRE1b regulated ER stress-triggered autophagy through its ribonuclease activity (Bao et al., 2018). This IRE1b-mediated autophagy is independent of its splicing target bZIP60 since autophagosome formation is unaffected in the *bzip60* mutant. Therefore, it is the RIDD activity but not the RNA splicing activity of IRE1b that is responsible for the activation of autophagy upon ER stress. 12 RIDD target genes were identified by transcriptomic analysis and three of their encoded proteins, BGLU21 (β-glucosidase 21), ROSY1/ML (interactor of synaptotagmin 1/MD2-related lipid recognition protein) and PR-14 (pathogenesis-related protein 14), are negative regulators of autophagy. In conclusion, IRE1b stimulates ER stress-triggered autophagy by degrading the mRNAs of several negative regulators of autophagy through RIDD (Bao et al., 2018). The regulatory mechanisms of autophagy by BGLU21, ROSY1/ML, and PR-14 are still unknown and remain to be elucidated in future research.

### Post-translational Regulation

Post-translational modifications are important in regulating protein activity by chemical modifying protein with functional groups, such as phosphate, methyl groups, and acetate (Deribe et al., 2010). Autophagy regulation at post-translational level is indispensable for plants to adapt to various environmental stresses. In this section, we focus on the phosphorylation, ubiquitination, and lipidation of ATG proteins which regulate the activity and duration of autophagy.

#### Phosphorylation

Protein phosphorylation is the most common post-translational modification in eukaryotes. Phosphorylation regulates autophagy activity through conformational changes in ATG protein structure, which causes protein activation or deactivation, thereby regulating their function (Jung et al., 2010; Noda and Fujioka, 2015). The phosphorylation level of ATG1 is important for autophagy initiation. In mammalian cells, AMP-activated protein kinase (AMPK) promotes autophagy by directly phosphorylating ULK1 (ATG1 homologue in animals) at Ser317 and Ser777 under glucose starvation (Kim et al., 2011). Conversely, the target of rapamycin (TOR) phosphorylates ULK1 at Ser757 to deactivate ULK1 under nutrient-rich conditions (Kim et al., 2011). Autophagy activity is also regulated by the phosphorylation level of other ATG proteins. For instance, AMPK phosphorylates BECN1 (ATG6 homologue in animals) at Thr388 to induce autophagy (Zhang D. et al., 2016). In addition, TOR negatively regulates autophagy through direct hyperphosphorylation of ATG13 in yeast (Kamada et al., 2010).

**FIGURE 1:**
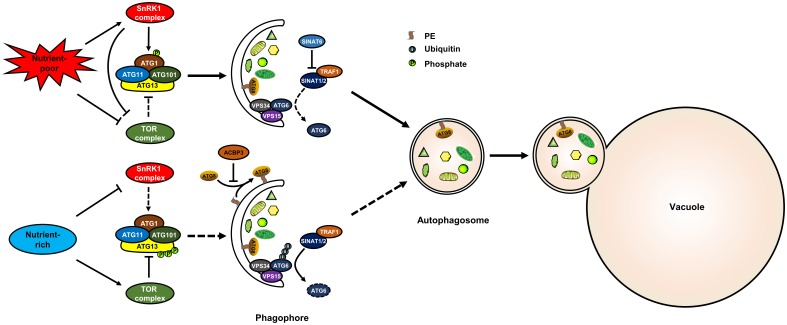
The post-translational regulation of autophagy in plants. The model shows known post-translational regulation of plant autophagy. Under nutrient-rich conditions, the activated TOR kinase phosphorylates ATG13 to inactivate the ATG1 complex, thereby suppressing autophagy; TRAF1s suppress autophagy by recruiting SINAT1/2 to ubiquitylate and degrade ATG6. Under nutrient-poor conditions, TOR is inhibited and SnRK1 is activated, and the activated SnRK1 induces autophagy by phosphorylating ATG1; SINAT6 disrupts the interaction between TRAF1s and SINAT1/2 to stabilize ATG6 and activate autophagy. ACBP3 disrupts autophagosome formation by competing with ATG8 for PE. SnRK1, sucrose non-fermenting 1–related kinase 1; TOR, target of rapamycin; ATG, autophagy-related; VPS, vacuolar protein sorting; TRAF, TNF receptor-associated factor; SINAT, SINA of *Arabidopsis thaliana*.

Target of rapamycin is a conserved Ser/Thr kinase that controls cell growth in all eukaryotes. TOR associates with the regulatory-associated protein of TOR (RAPTOR) and lethal with sec13 8 (LST8) to form a conserved TOR complex 1 (TORC1) in plants. Previous studies have indicated that TOR is a negative regulator of autophagy in plants (Liu and Bassham, 2010; Pu et al., 2017). Under nutrient-rich conditions, TOR is activated and, in turn, represses autophagy (Pu et al., 2017). ATG13 is also phosphorylated under such conditions (Suttangkakul et al., 2011). However, it was unknown whether TOR directly phosphorylates ATG13 in plant for a long time. Recently, results from large-scale phosphoproteomics showed that ATG13 is phosphorylated by TOR at S248, S397, S404, S406, S407, and S558 in *Arabidopsis* (Van Leene et al., 2019). Moreover, ATG13 interacts with RAPTOR through a plant TOS motif, and ATG13 lacking the TOS motif enhanced autophagy activity and could not be phosphorylated by TOR kinase (Son et al., 2018). These results indicate that TOR negatively regulates autophagy through direct phosphorylation of ATG13 in plants (Figure 1).

Previous research has suggested that the SnRK1 kinase (AMPK homologue in plants) is repressed by sugars, but activated under energy-deficient conditions, such as darkness, or biotic and abiotic stresses (Baena-Gonzalez et al., 2007). SnRK1 positively regulates autophagy through two different pathways: phosphorylation of ATG1 or phosphorylation of the TOR complex (Chen et al., 2017; Soto-Burgos and Bassham, 2017). In *Arabidopsis*, ATG1 is s phosphorylated upon nutrient deprivation (Suttangkakul et al., 2011). KIN10 is the most active protein of the SnRK1s and enhances the phosphorylation of ATG1 possibly through interacting with ATG1a (Chen et al., 2017) (Figure 1). Furthermore, KIN10 phosphorylates the TOR complex subunit RAPTOR (Nukarinen et al., 2016). KIN10-activated autophagy is blocked by TOR activation (Soto-Burgos and Bassham, 2017). These results suggest that KIN10 also regulates autophagy through the inhibition of TOR activity (Figure 1). However, the phosphorylation sites of ATG1 and RAPTOR that are recognized by SnRK1 have not been identified in *Arabidopsis*.

#### Ubiquitination

Generally, ubiquitination is a kind of post-translational protein modification in which proteins are labeled with ubiquitin and then recognized by the 26S proteasome for degradation (Kerscher et al., 2006). During autophagy, the stability and function of several core ATG components were highly influenced by ubiquitination (Xie et al., 2015). For example, TNF receptor-associated factor 6 (TRAF6) promotes autophagy by ubiquitination of BECN1 and ULK1 in mammalian cells (Shi and Kehrl, 2010; Nazio et al., 2013). TRAFs were previously identified as signaling adaptors and also function as E3 ubiquitin ligases. In *Arabidopsis*, TRAF proteins play dual roles in regulating autophagy by modulating ATG6 stability (Qi et al., 2017). Under nutrient-rich conditions, TRAF1a and TRAF1b recruit two RING finger E3 ligases, SINAT1/2 (SINA of *Arabidopsis thaliana*), to ubiquitylate and degrade ATG6, thereby suppressing autophagy (Figure 1). Upon starvation, the interaction between TRAF1a/1b and SINAT1/2 is disrupted by SINAT6, which leads to the stabilization of ATG6 and thus autophagy activation (Figure 1).

#### Lipidation

Lipidation is a post-translational modification by which proteins are covalently modified with specific lipids (Nadolski and Linder, 2007). In the process of autophagosome formation, ATG8 is lipidated by conjugating to phosphatidylethanolamine (PE) through a ubiquitin-like conjugation pathway (Avin-Wittenberg et al., 2012; Flick and Kaiser, 2012). Generally, lipidation of ATG8 is measured by western blot as a marker for autophagic activation (Klionsky et al., 2016). Recent studies have reported that ATG8 lipidation is enhanced by a range of stresses and hormones, such as pathogen infection (Kwon et al., 2013), drought (Wang et al., 2015), BL (Wang et al., 2019), and ACC (Zhu et al., 2018) treatments. Knockout of ATG5 or ATG7, the rate-limiting components of ATG8-PE conjugation, completely blocks ATG8 lipidation and autophagosome formation (Thompson et al., 2005; Phillips et al., 2008; Chung et al., 2010), whereas overexpression of ATG5 or ATG7 enhances ATG8 lipidation and therefore autophagosome formation (Minina et al., 2018). ATG4 is a cysteine protease and plays a dual role in ATG8 lipidation. On the one hand, ATG4 processes the carboxy-terminal Arg residue of the newly synthesized ATG8 for the exposure of the Gly residue, which is essential for ATG8 lipidation (Kirisako et al., 2000). On the other hand, ATG4 also delipidates ATG8 from the autophagosome membrane for recycling (Kirisako et al., 2000). In *Arabidopsis*, ATG4 mutation blocks the autophagy process because the ATG8s are unable to conjugate to PE (Yoshimoto et al., 2004). Interestingly, the abundance of PE can also influence plant autophagy activity. For example, acyl-CoA binding protein3 (ACBP3) *in Arabidopsis* can strongly bind PE, and therefore overexpression of ACBP3 disrupts autophagosome formation by competing with ATG8 for PE and modulating ATG8 stability (Xiao et al., 2010).

## Conclusion and Prospects

Great achievements have been made in characterizing the components of core autophagy machinery and the roles of autophagy in stress responses, development, and metabolism in plants. However, the regulatory mechanisms underlying plant autophagy remain largely unknown. As autophagy plays important roles in plant development and stress responses, fully understanding the complex network of regulatory factors that control autophagy processes will contribute to agronomic trait improvement by manipulating autophagy in crops. Several autophagy regulators have been identified and characterized at the transcriptional, post-transcriptional, and post-translational levels in plants. However, there are fewer regulators of plant autophagy than there are for yeast and animals, and probably many more that remain unidentified in plants. There is a large gap in the literature regarding the post-transcriptional regulation of autophagy in plants, such as the process in which miRNA targets *ATG* genes to repress gene expression. In addition, modifications of ATG proteins at post-translational level, such as acetylation, are yet to be determined in plants. Protein acetylation fine controls mammalian autophagy at multiple levels, including ATG proteins and regulatory proteins (Banreti et al., 2013). Therefore, identification of novel regulators involved in the regulation of plant autophagy remains a critical and challenging subject for future research.

## Author Contributions

WH and LC conceived the idea. MY and FB wrote the first draft. LC critically revised the manuscript. All the authors read and approved the final content.

## Conflict of Interest Statement

The authors declare that the research was conducted in the absence of any commercial or financial relationships that could be construed as a potential conflict of interest.
